# Role of RNA Biogenesis Factors in the Processing and Transport of Human Telomerase RNA

**DOI:** 10.3390/biomedicines10061275

**Published:** 2022-05-30

**Authors:** Tatiana Pakhomova, Maria Moshareva, Daria Vasilkova, Timofey Zatsepin, Olga Dontsova, Maria Rubtsova

**Affiliations:** 1 Department of Chemistry, A. N. Belozersky Institute of Physicochemical Biology, Lomonosov Moscow State University, Moscow 119991, Russia; tatiana.pakhomova@chemistry.msu.ru (T.P.); moshareva.m@gmail.com (M.M.); dvasilkova@gmail.com (D.V.); olga.a.dontsova@gmail.com (O.D.); 2 Shemyakin-Ovchinnikov Institute of Bioorganic Chemistry of the Russian Academy of Sciences, Moscow 117997, Russia; tsz@yandex.ru; 3 Center of Life Sciences, Skolkovo Institute of Science and Technology, Skolkovo, Moscow 121205, Russia

**Keywords:** telomerase, processing, transcription, biogenesis, Integrator complex, RNA transport, RNA exosome

## Abstract

Telomerase RNA has long been considered to be a noncoding component of telomerase. However, the expression of the telomerase RNA gene is not always associated with telomerase activity. The existence of distinct *TERC* gene expression products possessing different functions were demonstrated recently. During biogenesis, hTR is processed by distinct pathways and localized in different cell compartments, depending on whether it functions as a telomerase complex component or facilitates antistress activities as a noncoding RNA, in which case it is either processed in the mitochondria or translated. In order to identify the factors responsible for the appearance and localization of the exact isoform of hTR, we investigated the roles of the factors regulating transcription DSIF (Spt5) and NELF-E; exosome-attracting factors ZCCHC7, ZCCHC8, and ZFC3H1; ARS2, which attracts processing and transport factors; and transport factor PHAX during the biogenesis of hTR. The data obtained revealed that ZFC3H1 participates in hTR biogenesis via pathways related to the polyadenylated RNA degradation mechanism. The data revealed essential differences that are important for understanding hTR biogenesis and that are interesting for further investigations of new, therapeutically significant targets.

## 1. Introduction

Telomerase RNA (TR) is a component of the telomerase complex [1] that maintains the length of the telomere regions located at the ends of linear eukaryotic chromosomes [2]. Telomerase is active in cells with high proliferative rates, such as stem cells, germ cells, and cancer cells, and is inactive in the majority of somatic cells [3]. Telomerase inactivation occurs because of the silencing of telomerase reverse transcriptase (*TERT*) expression [4]. However, *hTERC* expression remains constitutive in most somatic cells [5]. TR has long been considered to be a noncoding RNA component of telomerase. Recently, it was demonstrated that the precursor of hTR encodes a protein, hTERP [6], that protects cells against drug-induced apoptosis and that is involved in autophagy regulation via the activation of AMPK-mediated TSC2 Ser1387 phosphorylation. hTR may be processed in mitochondria to produce short TERC-53 RNA [7], which regulates senescence [8]. The Integrator complex [9], cap binding complex (CBC), and RNA exosomes [10] are involved in the biogenesis of hTR. The depletion of these factors affects processing, resulting in the accumulation of the 3′-extended form of the hTR transcript. The existence of distinct forms of functional hTR transcripts and their presence in both the nucleus and cytoplasm suggest that the regulation of primary transcript processing and transport plays an important role in the function and cellular fate of hTR.

In eukaryotic cells, RNA biogenesis involves multiple tightly coordinated stages: transcription, capping, splicing, 3′-end processing, and transport. Disturbing the coordination of these distinct stages results in the formation of aberrant, non-functional transcripts that undergo fast turnover. RNA polymerase II (RNAPII) synthesizes distinct classes of RNAs, and additional factors determine the specific co-transcriptional processing of coding and noncoding transcripts. First, capping coincides with RNAPII pausing, which is facilitated by NELF and DSIF soon after transcription initiation [11,12]. It has been demonstrated that the Integrator complex is recruited by DSIF and NELF to pause RNAPII, and the subunits of the complex also regulate the activity of transcriptional machinery [13]. CBC is attracted to mRNA through NELF-E [14]. The effective elongation of transcription is promoted by the phosphorylation of the C-terminal domains of RNAPII, DSIF, and NELF [15,16,17]. The involvement of the Integrator, DSIF, and NELF in the 3′-end processing of snRNAs and in replication-dependent histone mRNAs has been demonstrated previously [13,14]. It has been proposed that RNAPII pausing is necessary to correct 3′-end processing and that NELF prevents aberrant polyadenylation by inhibiting the recruitment of the cleavage stimulation factor [13]. ARS2 substitutes NELF-E in the complex formed with CBC at the termination of transcription and attracts the PHAX protein, which mediates the transport of correct, newly synthesized snRNA transcripts from the nucleus to the cytoplasm or through the nucleoplasm [18,19]. Aberrant transcripts are targeted for exosomal degradation by the interaction of RNA exosome components with ARS2 [18]. 

RNA exosomes play a main role in the correct processing or degradation of hTR [10,20,21]. The core catalytic complex of exosomes is targeted by the RNA-binding adaptor proteins that are specific to different types of transcripts [22]. Three distinct RNA exosome complexes: NEXT, TRAMP, and PAXT, process the transcripts synthesized by RNA polymerase II. ZCCHC7 attracts the TRAMP complex that degrades certain RNA substrates after the addition of an unstructured oligo(A)-tail at the 3′-end. TRAMP is known as a complex that produces mature hTR. ZCCHC8 promotes hTR degradation that is not targeted to the TRAMP by NEXT [10]. The ZFC3H1 component of the PAXT exosome complex was recently identified as a factor that competes with PABPN1 for the binding poly(A)-tails. Its role in hTR processing and degradation has not yet been investigated. However, the involvement of PABPN1 in the regulation of hTR maturation was demonstrated earlier [20]. PABPN1 protects oligoadenylated hTR transcripts and assists in PARN-dependent hTR maturation. The absence of PABPN1 results in hTR degradation. 

hTR is processed by distinct pathways and is localized in different cell compartments, depending on whether it functions as a telomerase complex component in nuclear activities or facilitates antistress activities, where it is either processed in the mitochondria or translated in the cytoplasm. In order to identify the factors that are responsible for the appearance and localization of the exact isoform of hTR, we investigated the role of factors regulating transcription DSIF (Spt5) and NELF-E; exosome-attracting factors ZCCHC7, ZCCHC8, and ZFC3H1; ARS2, which attracts processing and transport factors; and the transport factor PHAX in hTR biogensis.

## 2. Materials and Methods

### 2.1. Cell Culture

Human HEK293T cells were grown in DMEM/F12 medium supplemented with Glutamax (Thermo Fisher Scientific, Waltham, MA, USA), 10% fetal bovine serum (FBS), 100 U/mL penicillin, and 100 μg/mL streptomycin at 37 °C and 5% CO_2_. Cultures were examined under an inverted microscope to determine confluency and viability. The cells were confirmed to be negative for mycoplasma contamination.

### 2.2. RNAi Analysis

After 48 h of cell treatment, siRNA knockdown was performed, and the working concentration was determined. For siRNA knockdown, the cells were treated three times (at 2 d intervals) with siRNA, as listed in Table A1, using Lipofectamine RNAiMAX (Thermo Fisher Scientific, Waltham, MA, USA) according to the manufacturer’s instructions. The primary treatment was performed as reverse transfection, and the secondary and tertiary treatments were performed as forward transfection. The knockdown efficiency was determined by RT-qPCR using the primers listed in Table A2 and by Western blotting with antibodies specific to the targets: anti-SPT5 antibody (EPR5145(2)) (ab126592), anti-ZCCHC7 antibody (ab104503), anti-ZCCHC8 antibody (EPR13612) (ab181152), anti-ARS2 antibody (ab192999), anti-PHAX Antibody (F-1) sc-398147, anti-ZFC3H1 Antibody (PA5-103755), and anti-NELF-E (EPR11600) (ab170104).

### 2.3. Nuclear and Cytoplasmic RNA Quantification

Fractions of cytoplasmic and nuclear RNA were prepared according to Wang et al. [23]. Approximately 5 × 10^6^ cells were used for total RNA purification and nuclear and cytoplasmic fractionation. The cells were washed with PBS three times and scraped with a cell scraper. The obtained cells were centrifuged and washed with RSB (10 mM Tris-HCl, pH 7.4; 3 mM MgCl_2_; and 10 mM NaCl). The cytoplasmic fraction was extracted using RSBG40 (10 mM Tris-HCl, pH 7.4; 3 mM MgCl_2_; 10 mM NaCl; 10% glycerol; Nonidet-P40; and 0.5 mM DTT). Nuclei were washed with RSBG40 and a 1/10 volume of detergent (3.3% *wt*/*wt* sodium deoxycholate and 6.6% *v*/*v* Tween 20). The pellet was used as the nuclear fraction. RNA and protein were extracted using TRIzol (Invitrogen, Waltham, MA, USA) according to the manufacturer’s instructions. The Western blot analysis of fibrillarin (CST (2639S), 1:1000) and α-tubulin (Abcam, Cambridge, UK) (ab18251), 1:2000) showed the separation of the nuclear and cytoplasmic fractions. Total, cytoplasmic, and nuclear RNA were treated with DNAse I followed by reverse transcription with random primers, and the obtained cDNA was used for PCR. In the cytoplasmic and nuclear fractions, hTR quantification was performed with hTR-specific primers, GAPDH mRNA, and GAPDH pre-mRNA (Table A2). To confirm the specificity of the primers, the RT-qPCR products were sequenced.

### 2.4. Quantitative Real-Time PCR 

RNA was isolated using a PureLink RNA Mini Kit (Thermo Fisher Scientific, Waltham, MA, USA) according to the manufacturer’s instructions. The RNA was reverse transcribed into cDNA using the Maxima First Strand Kit cDNA Synthesis for RT-qPCR (Thermo Fisher Scientific, Waltham, MA, USA) according to the manufacturer’s protocols. Quantitative PCR was performed on the cDNA in triplicate using primers specific to hTR PCR (Table A2). A reaction mixture containing SYBR Green Nucleic Acid Stain (Invitrogen) was used along with the CFX96 Real-Time system (Bio-Rad, Hercules, CA, USA). The PCR cycle parameters were as follows: 95 °C for 10 min, 35 cycles of denaturation at 95 °C for 30 s, annealing at 58 °C for 30 s, and extension at 72 °C for 40 s. Relative gene expression was determined using the 2^−ΔΔCt^ method [24]. The relative expression level of the RNA was calibrated using the geometric mean of the GAPDH mRNA and U2 snRNA levels to minimize experimental variation. Minus–RT controls, established by omitting the RT enzyme in a mock reaction to rule out DNA contamination, were used in every RT-qPCR analysis.

### 2.5. Statistical Analysis

Statistical analysis was performed using GraphPad Prism 7.0 software (GraphPad, La Jolla, CA, USA). Statistical significance was determined using one-way and two-way analysis of variance (ANOVA), and differences between the analyzed samples were determined using Sidak’s or Dunnett’s multiple comparison tests. Each experiment was repeated at least three times.

## 3. Results

To investigate the role of DSIF, NELF, ARS2, PHAX, and ZCCHC7, ZCCHC8, and ZFC3H1 in the biogenesis and transport of hTR, we performed the siRNA-mediated knockdown of selected genes and analyzed the total amounts of hTR and its precursor in whole cell lysates, cytoplasmic fractions, and nuclear fractions. U2 snRNA was used as a control because its transcription is Integrator-dependent [13], and it is transported to the cytoplasm to form an RNA–protein complex [25] that returns to the nucleus. In contrast to hTR, U2 snRNA is not translated.

First, we developed and validated the subcellular fractionation procedure to obtain clear cytoplasmic and nuclear lysates suitable for further analysis. The nuclear and cytoplasmic fractions were separated according to a previously described protocol for mRNA-level RT-qPCR quantification [23], and modifications were made to improve lysis and the separation of the nuclear and cytoplasmic fractions. To lyse the cells, we used original buffer containing either 0.5% or 1% Nonidet P-40 (full composition described in the Section 2). After a short incubation period on ice (3 min), the samples were centrifuged, and the supernatants were retained as the cytoplasmic fractions. The pellets were resuspended in lysis buffer containing 3.3% sodium deoxycholate and 6.6% Tween 40, incubated for 5 min on ice, and centrifuged. The obtained pellets were retained as nuclear fractions. To assess the quality of the separation, we measured the RNA concentration using NanoDrop. We found that only the buffer containing 1% Nonidet P-40 lysed the cells completely (Table A3). 

**Figure A1:**
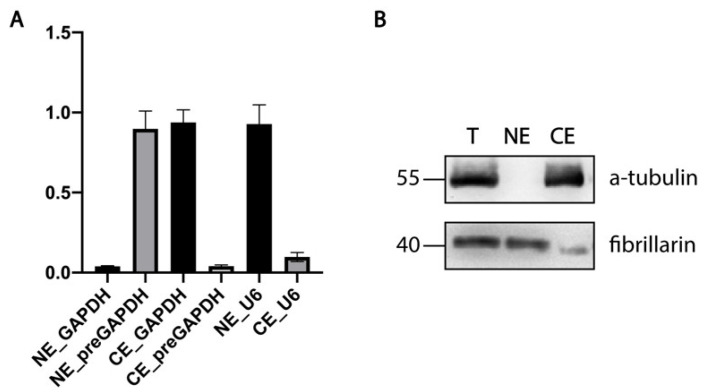
The quality of cellular extract separation was confirmed by RNA and protein analysis. (**A**) The RNA purified from the nuclear (NE) and cytoplasmic (CE) fractions of HEK293T cells was subjected to RT-qPCR to determine the amount of unspliced GAPDH (preGAPDH) and spliced GAPDH transcripts and U6 snRNA. (**B**) The total cellular protein (T) and proteins extracted from the CE and NE fractions were analyzed by Western blotting with the specified antibodies.

To confirm the quality of subcellular separation, we determined the amount of spliced and unspliced GAPDH mRNA and U6 snRNA in the cytoplasmic and nuclear fractions using RT-qPCR (Figure A1A) as well as the amount of α-tubulin and fibrillarin by Western blotting (Figure A1B). We observed an accumulation of unspliced preGAPDH mRNA, U6 snRNA, and fibrillarin in the nuclear fraction and spliced GAPDH mRNA and α-tubulin in the cytoplasmic fraction, confirming the quality of the lysate separation with 1% Nonidet P-40 (Figure A1). Therefore, we used the modified procedure described above for the separation of the cytoplasmic and nuclear fractions for further analysis.

**Figure A2:**
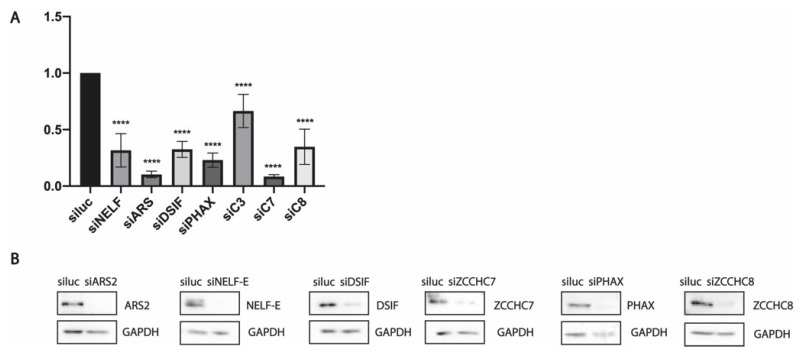
siRNA efficiency. (**A**) The relative levels of the mRNAs targeted with the specified siRNAs after the knockdown were revealed by RT-qPCR. (**B**) Levels of proteins targeting mRNAs with specified siRNAs were analyzed by Western blotting with specific antibodies. **** *p* < 0.0001 by Dunett’s multiple comparison test.

To investigate the factors affecting RNA biogenesis and transport in telomerase RNA processing, we performed the siRNA-mediated knockdown of *DSIF*, *NELF*, *ARS2*, *PHAX*, *ZCCHC7*, *ZCCHC8*, and *ZFC3H1* using the previously described siRNAs (Table A2). First, we determined the efficacy of the siRNAs in inhibiting the expression of specific genes. Briefly, the siRNAs were transfected into HEK293T cells at 3, 10, and 30 nM concentrations, and the mRNA levels of the targeted genes were analyzed by RT-qPCR with specific primers (Table A2) after incubation for 48 h (Figure A2A). The depletion of the targeted proteins was analyzed by Western blotting (Figure A2B). Firefly luciferase-specific siRNA, which is not expressed in HEK293T cells, was used as a scrambled control. Further experiments were performed using siRNAs at the optimal concentration to efficiently inhibit gene expression. 

**Figure 1:**
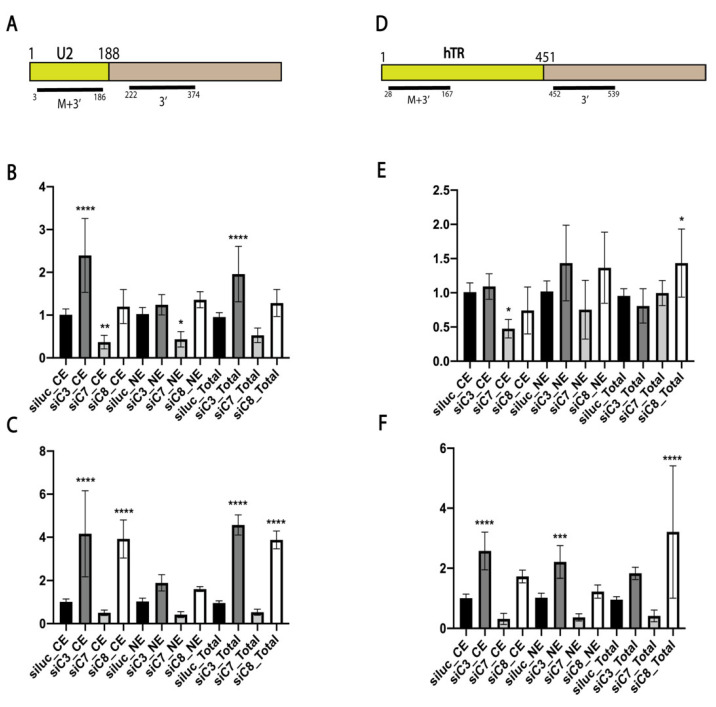
The role of RNA exosome-associated factors in processing and transport of U2 snRNA and hTR. (**A**) Scheme illustrating regions of U2 snRNA that were amplified as total (M+3′) and unmatured transcript (3′). (**B**) Histograms demonstrating the relative amount of total (M+3′) U2 snRNA transcripts in the cytoplasmic (CE) and nuclear (NE) fractions and in the whole cell extract (Total) after the knockdown of the specified RNA biogenesis factor. siluc—control knockdown performed with siRNA specific to Firefly luciferase mRNA; siC3*—*knockdown performed with siRNA specific to ZFC3H1; siC7*—*knockdown performed with siRNA specific to ZCCHC7; siC8*—*knockdown performed with siRNA specific to ZCCHC8. (**C**) Histograms demonstrating the relative amount of premature (3′) U2 snRNA transcripts in the cytoplasmic (CE) and nuclear (NE) fractions and in the whole cell extract (Total) after the knockdown of the specified RNA biogenesis factor. (**D**) Scheme illustrating regions of hTR that were amplified as total (M+3′) and unmatured transcripts (3′). (**E**) Histograms demonstrating the relative amount of total (M+3′) hTR transcripts in the cytoplasmic (CE) and nuclear (NE) fractions and in the whole cell extract (Total) after the knockdown of the specified RNA biogenesis factor. (**F**) Histograms demonstrating the relative amount of the premature (3′) hTR transcripts in the cytoplasmic (CE) and nuclear (NE) fractions and in the whole cell extract (Total) after the knockdown of the specified RNA biogenesis factor. * *p* < 0.1, ** *p* < 0.01, *** *p* < 0.001, and **** *p* < 0.0001 by Dunett’s multiple comparison test.

In order to perform the knockdown of *DSIF*, *NELF*, *ARS2*, *PHAX*, *ZCCHC7*, *ZCCHC8*, and *ZFC3H1* or double knockdowns, we incubated the cells with individual siRNAs or a mix of two siRNAs for 6 d. Whole cell lysates and cytoplasmic and nuclear fractions were subjected to RT-qPCR analysis to determine the total hTR and U2 RNA transcript levels and 3′-end extended precursors levels. To determine the total transcript and 3′-extended precursor levels, we used a random hexamer primer for cDNA synthesis, which was further amplified with different sets of primers specific to distinct regions of the analyzed transcript. The schemes in Figure 1A,D illustrate the regions of U2 snRNA and hTR that were amplified as total (M+3′) and premature 3′-extended (3′) transcripts. 

First of all, we noted that the depletion of different RNA-binding protein adaptors of exosomes demonstrated similar effects during the processing of U2 snRNA and hTR (Figure 1B,C,E,F). We observed the accumulation of both the total and 3′-extended transcripts of U2 snRNA (Figure 1B,C) and the 3′-extended hTR transcripts (Figure 1F) when ZCCHC8 and ZFC3H1 were depleted, and the amounts of U2 snRNA (Figure 1B,C) and unprocessed hTR (Figure 1F) decreased after the knockdown of *ZCCHC7*. 

**Figure 2:**
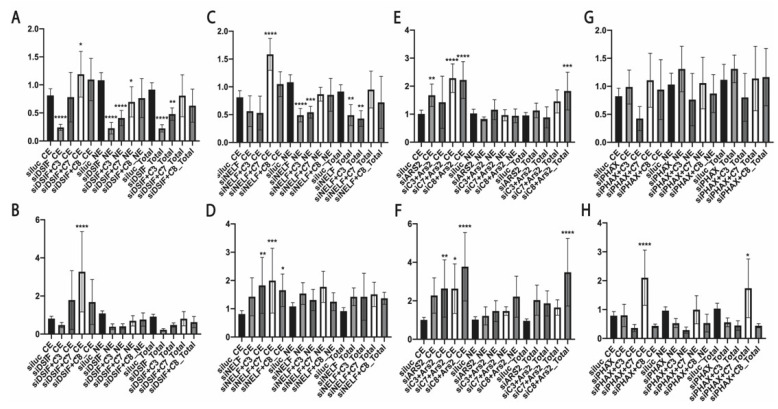
The role of RNA biogenesis factors in hTR processing and transport. (**A**,**C**,**E**,**G**) Histograms demonstrated the relative amount of total (M+3′) hTR transcripts in the cytoplasmic (CE) and nuclear (NE) fractions and in the whole cell extract (Total) after the knockdown of the specified RNA biogenesis factor. (**B**,**D**,**F**,**H**) Histograms demonstrating the relative amount of prematured (3′) hTR transcripts in the cytoplasmic (CE) and nuclear (NE) fractions and in the whole cell extract (Total) after the knockdown of the specified RNA biogenesis factor. siluc*—*control knockdown performed with siRNA specific to Firefly luciferase mRNA; siC3*—*knockdown performed with siRNA specific to ZFC3H1; siC7*—*knockdown performed with siRNA specific to ZCCHC7; siC8*—*knockdown performed with siRNA specific to ZCCHC8. * *p* < 0.1,** *p* < 0.01, *** *p* < 0.001, and **** *p* < 0.0001 by Dunett’s multiple comparison test.

**Figure 3:**
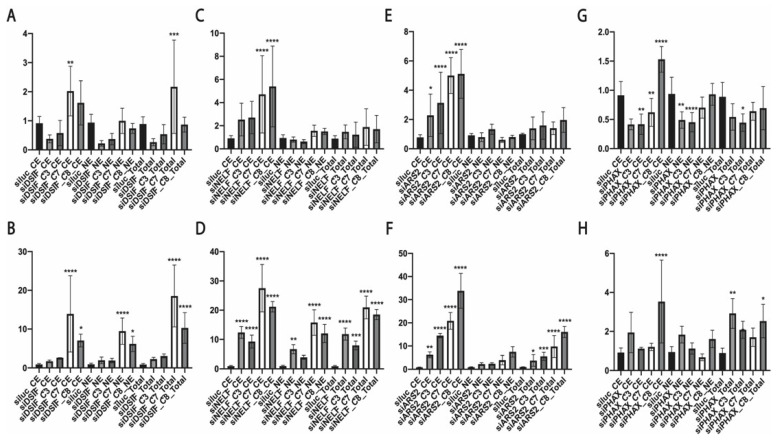
The role of RNA biogenesis factors in processing and transport of U2 snRNA. (**A**,**C**,**E**,**G**) Histograms demonstrating the relative amount of total (M+3′) U2 snRNA in the cytoplasmic (CE) and nuclear (NE) fractions and in the whole cell extract (Total) after the knockdown of the specified RNA biogenesis factor. (**B**,**D**,**F**,**H**) Histograms demonstrating the relative amount of premature (3′) U2 snRNA transcripts in the cytoplasmic (CE) and nuclear (NE) fractions and in the whole cell extract (Total) after the knockdown of the specified RNA biogenesis factor. siluc*—*control knockdown performed with siRNA specific to Firefly luciferase mRNA; siC3*—*knockdown performed with siRNA specific to ZFC3H1; siC7*—*knockdown performed with siRNA specific to ZCCHC7; siC8*—*knockdown performed with siRNA specific to ZCCHC8. * *p* < 0.1, ** *p* < 0.01, *** *p* < 0.001, and **** *p* < 0.0001 by Dunett’s multiple comparison test.

We observed decreased hTR and U2 snRNA levels after the depletion of DSIF that was recovered when ZCCHC7, ZCCHC8, and ZFC3H1 were co-depleted (Figure 2A,B and Figure 3A,B). The knockdown of *NELF-E* resulted in decreased levels of total hTR (Figure 2C), but the levels of unprocessed transcripts increased slightly (Figure 2D). The total hTR level was restored when ZCCHC7 or ZCCHC8 were co-depleted (Figure 2C). NELF-E depletion resulted in the significant accumulation of unprocessed U2 snRNA transcripts in both the cytoplasmic and nuclear fractions (Figure 3D), but the level of total U2 snRNA transcripts only increased in the cytoplasmic fraction (Figure 3C) predominantly. 

*ARS2* knockdown promoted the accumulation of total and unprocessed hTR in the cytoplasmic fraction that was accelerated when ZCCHC8 was co-depleted (Figure 2F). The dramatic accumulation of U2 snRNA in the cytoplasmic fraction (Figure 3E,F) was observed after the knockdown of *ARS2* and *ZCCHC7* or *ZCCHC8*. 

The most interesting data were obtained when inhibiting *PHAX* expression (Figure 2G,H and Figure 3G,H). We did not observe significant effects at the total hTR (Figure 2G) and U2 snRNA (Figure 3G) levels, but the level of unprocessed hTR transcript decreased in the nuclear fraction (Figure 2H). The co-depletion of PHAX and ZFC3H1 resulted in a decreased level of total and 3′-extended hTR transcripts in all fractions (Figure 2G,H). The co-depletion of ZCCHC7 led to the accumulation of unprocessed hTR (Figure 2H). The same effect was observed for unmature U2 snRNA transcripts that had accumulated when PHAX was depleted (Figure 3G). The accumulation of total U2 snRNA in the cytoplasmic fraction was observed in the double knockdown of *PHAX* and *ZCCHC8* (Figure 3G). However, the accumulation of unprocessed U2 snRNA was detected when PHAX was depleted alone or in combination with ZCCHC8 (Figure 3H). 

**Figure 4:**
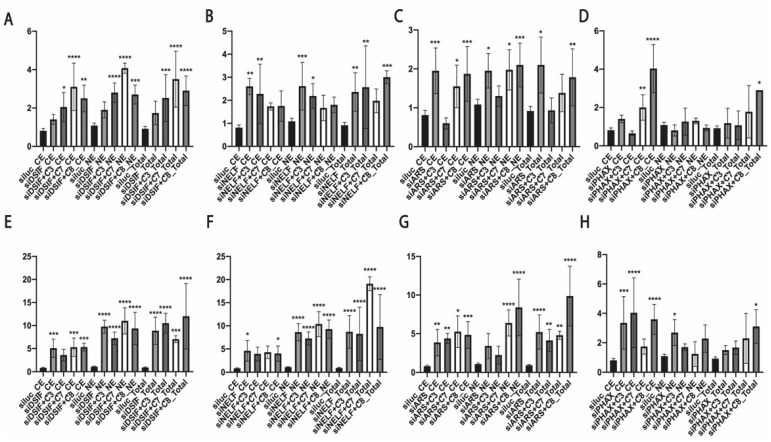
Processing efficiency of hTR and U2 snRNA. To compare the processing efficiency, the relative amount of premature transcripts (3′) was normalized to the relative amount of total transcripts (M+3′). (**A**–**D**) Histograms demonstrating the efficiency of processing hTR in the cytoplasmic (CE) and nuclear (NE) fractions and whole cell extract (Total) after the knockdown of the specified RNA biogenesis factor. (**E**–**H**) Histograms demonstrating the efficiency of processing U2 snRNA in the cytoplasmic (CE) and nuclear (NE) fractions and in the whole cell extract (Total) after the knockdown of the specified RNA biogenesis factor. siluc*—*control knockdown performed with siRNA specific to Firefly luciferase mRNA; siC3*—*knockdown performed with siRNA specific to ZFC3H1; siC7*—*knockdown performed with siRNA specific to ZCCHC7; siC8*—*knockdown performed with siRNA specific to ZCCHC8. * *p* < 0.1, ** *p* < 0.01, *** *p* < 0.001, and **** *p* < 0.0001 by Dunett’s multiple comparison test.

To compare the efficacy of the 3′-end processing in the investigated transcripts, we normalized the amount of 3′-extended transcripts (3′) to the number of total transcripts (M+3′) (Figure 4). DSIF depletion alone affected hTR processing slightly (Figure 4A). *NELF-E* knockdown alone demonstrated the accumulation of unprocessed hTR, and the co-depletion of RNA exosome-attracting factors did not demonstrate any additional effects (Figure 4B). *ARS2* knockdown resulted in the inhibition of hTR processing, and the co-depletion of ZFC3H1 restored hTR maturation to wild type levels (Figure 4C). The co-depletion of PHAX with ZCCHC8 stimulated the accumulation of unprocessed hTR transcripts in the cytoplasmic fraction and demonstrated total maturation inhibition (Figure 4D). 

We observed that the knockdown of *DSIF*, *NELF-E*, *ARS2*, and *PHAX* stimulated the accumulation of unprocessed forms of U2 snRNA (Figure 4E–H). The co-depletion of exosome-attracting factors with DSIF did not have a significant influence on the processing of U2 snRNA (Figure 4E–H). The co-depletion of ZCCHC7 with NELF-E promoted the total accumulation of unprocessed forms of U2 snRNA (Figure 4F). The double knockdown of *ARS2* with *ZCCHC7* demonstrated the accumulation of unprocessed forms of U2 snRNA in the nuclear fraction (Figure 4G), and we observed significant processing inhibition when ARS2 was co-depleted with ZCCHC8 (Figure 4G). 

## 4. Discussion

Eukaryotic cells produce various types of RNA that follow certain processing/decay and/or transport pathways. Proper sorting into appropriate pathways determines the fate of the nascent transcript, and sorting is carried out according to its particular function in the cell. TR represents a case in which a particular RNA has at least two important functions: a component of the telomerase complex, which participates in telomere length maintenance in the nucleus, and a messenger RNA for hTERP synthesis in the cytoplasm. The complicated functions of TR suggest that TR biogenesis is subjected to specific regulations at different stages, such as transcription, processing, transport, and turnover, that defines its pathway. 

DSIF and NELF work as attractive factors that facilitate Integrator binding with the transcriptional elongating complex [26] and regulate the transcription and processing of snRNAs and hTR. DSIF and NELF pause the RNAPII at the start of transcription, which is necessary to attract CBC via NELF [14]. CBC functions as a platform for the recruitment of ARS2, PHAX, and other RNA biogenesis factors [18,27]. ARS2 attracts exosomes by interacting with different RNA-binding Zn-finger proteins such as ZFC3H1, ZCCHC7, and ZCCHC8, resulting in transcript degradation or proper maturation. ZFC3H1 competes with PABPN1 to bind with polyadenylated RNAs, ZCCHC7 attracts the TRAMP–exosome complex that matures hTR, and ZCCHC8 participates in the degradation of targeted RNAs by the NEXT*–*exosome complex. PHAX facilitates RNA transport from the nucleus to the cytoplasm or the intranuclear localization of some special types of RNAs, such as snoRNAs [19]. 

In this study, we demonstrated that *DSIF* knockdown has an inhibitory effect on the transcription of U2 snRNA, in agreement with previously published data [13]. *NELF-E*, *PHAX,* and *ARS2* knockdown resulted in the accumulation of an unprocessed form of U2 snRNA but it did not influence the amount of U2 snRNA in the nuclear and total extracts. The co-depletion of ZFC3H1 and ZCCHC7 did not affect the processing of U2 snRNA, but the co-depletion of ZCCHC8 with DSIF stimulated the accumulation of U2 snRNA. *DSIF* knockdown resulted in the inhibition of U2 snRNA processing alone and in combination with RNA exosome-attracting factors. We observed additional effects during U2 snRNA processing when ZCCHC7 or ZCCHC8 were co-depleted with other factors and did not detect the influence of ZFC3H1 on U2 snRNA maturation. These data confirm that the TRAMP and NEXT exosomes participate in U2 snRNA processing via the poly(A)-independent pathway.

We demonstrated that the knockdown of *DSIF* results in decreased levels of mature hTR transcripts, and the co-depletion of any exosome targeting protein increased the amount of hTR in cells. As such, DSIF is necessary for hTR transcription, and its deficiency results in the synthesis of aberrant transcripts that are degraded rapidly by exosomes. NELF-E deficiency increased the transcription rate via the more effective production of 3′-extended hTR transcripts that are degraded by the TRAMP and NEXT exosomes, resulting in decreased levels of mature hTR. The co-depletion of ZFC3H1 with NELF-E did not restore the hTR level to the control level. We propose that NELF deficiency stimulates the production of aberrant hTR transcripts that are used as a target by TRAMP and NEXT exosome complexes. We could not exclude the involvement of the PARN-mediated degradation of aberrant polyadenylated hTR transcripts that may be enriched due to the conditions introduced by inhibited *NELF-E* expression.

ARS2 deficiency did not influence the level of mature hTR and increased the level of the 3′-extended form of hTR. We observed hTR enrichment in the cytoplasmic fraction under conditions when ARS2 was deficient. The co-depletion of ZCCHC8 increased the level of the 3′-extended form of the hTR transcript. We propose that in the absence of ARS2, some unknown transport factor is attracted to the hTR transcripts and promotes export from the nucleus to the cytoplasm more efficiently.

PHAX deficiency does not influence the level of mature hTR. The co-depletion of ZFC3H1 decreased the amount of mature and 3′-extended hTR transcripts, and the co-depletion of ZCCHC7 stimulated the accumulation of 3′-extended hTR transcripts, especially in the cytoplasmic fraction. 

The depletion of exosome-targeting proteins ZFC3H1 and ZCCHC8 resulted in the accumulation of the 3′-extended form of hTR and decreased levels of this transcript were observed when *ZCCHC7* expression was inhibited. We can conclude that the PAXT and NEXT exosome complexes are involved in the degradation of excessive amounts of hTR, while the TRAMP complex participates in the maturation of native transcripts to mature hTR. 

**Figure 5:**
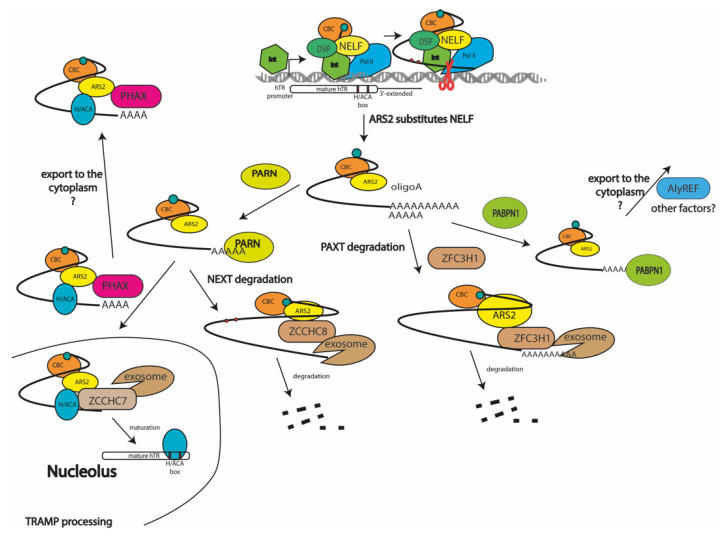
Scheme illustrating hTR processing and turnover pathways.

The data obtained here, and the previously published data, allow us to propose a scheme for hTR processing and turnover in cells (Figure 5). RNA-pol II accompanied by DSIF and NELF produces different oligoadenylated forms of primary hTR transcripts. The NELF protein should be substituted by ARS2, which works as a platform to coordinate the processing and degradation pathways. PABPN1 [20] is associated with oligoadenylated transcripts and prevents them from being degraded by exosomes or PARN. Transcripts that contain a shorter oligoA tail are rapidly degraded by the PAXT exosome complex or processed by PARN to be degraded by NEXT or matured by TRAMP. The pool of oligoA-tailed hTR transcripts associated with PABPN1 may be exported to the cytoplasm, where translation with hTERP protein production or import to the mitochondria occur.

The CBC*–*PHAX*–*CRM1 complex transports U snRNAs from the nucleus to the cytoplasm and through nucleus, where it targets the U snRNAs to the nucleolus or Cajal bodies for further processing [19,28]. The association of hTR with PHAX was demonstrated previously [19]. It is known that mature mRNAs are transported from the nucleus by the CBC*–*TREX*–*NXF1 complex [29]. Aly/REF associated with NXF1 directly binds to the target mRNA to allow docking with nucleoporins [30]. Aly/REF competes with ZFC3H1 for the mRNA binding that prevents mRNA from targeting the RNA exosome [31]. We hypothesize that in the absence of both the PHAX transport factor and TRAMP complex, the 3′-extended hTR transcript accumulates and redirects to an alternative transport pathway that is inherent to mRNA. In the absence of ZFC3H1, the accumulated hTR precursor may be more efficiently degraded by the NEXT complex, resulting in decreased hTR levels. As such, our data allow us to conclude that PHAX is involved in intranuclear transport and hTR localization. PHAX regulates the processing of mature hTR and is necessary for the general appearance of the active telomerase complex, but we cannot exclude the participation of PHAX in the nuclear export of a small portion of hTR under special conditions. The components of the CBC*–*TREX*–*NXF1 transport complex may be very attractive targets for the further analysis of hTR biogenesis.

Recent data concerning the multiple functions of hTR raise questions about the mechanisms of 3’-end processing and localization in different cellular compartments. The present study compared the roles of several of the RNA biogenesis factors that are involved in the processing and transport of hTR. The data revealed essential facts that are important for understanding hTR biogenesis and that are interesting for further investigations of new, therapeutically significant targets.

## Data Availability

The data that support the findings of this study are available from the corresponding author upon reasonable request.

## Appendix A

**Table A1:** siRNAs used in this study.

Target	Sequences
PHAX_1	GUAUCAGCGAGGAACAAAUUAdTsdT
PHAX_2	GAGUAUAUAGCACAGGAUUUAdTsdT
NELF	GAUGGAGUCAGCAGAUCAGdTsdT
DSIF	GAACUGGGCGAGUAUUACAdTsdT
Ars2_1	CCCGUCGUGUCCGCAACAUAAdTsdT
Ars2_2	CCGAAGAAGCACUUAAAGAAAdTsdT
Luc	CUUACGCUGAGUACUUCGATsT

**Table A2:** Oligonucleotides used in this study for reverse transcription and polymerase chain reaction.

Name	Sequences	Note
GAPDH_Fw	TGCACCACCAACTGCTTAGC	Spliced mRNA *GAPDH*
GAPDH_Rv	GGCATGGACTGTGGTCATGAG	
preGAPDH_Fw	CCACCAACTGCTTAGCACC	Unspliced mRNA *GAPDH*
preGAPDH_Rv	CTCCCCACCTTGAAAGGAAAT	
PHAX_Fw	ACAGGCTAGGGAACAGACCA	Total mRNA *PHAX*
PHAX_Rv	TCACTACTCGGGCTATCAGGT	
ARS2_Fw	ATTTCTTCCTGGCGCACAAA	Total mRNA *ARS2*
ARS2_Rv	ATCAAACCAGCCAGTCTCCA	
DSIF_Fw	AAGATGCCACAGAGTCCACG	Total mRNA *DSIF*
DSIF_Rv	CCTCCCATAGGTCGAGGTCA	
NELF-E_Fw	TTCAGCACTTGGGATTGGGG	Total mRNA *NELF-E*
NELF-E_Rv	TCCAGATCCTTTTGGGGCAA	
hTR_ M+3′_Fw	GTGGTGGCCATTTTTTGTCTAAC	Total hTR
hTR_ M+3′_Rv	TGCTCTAGAATGAACGGTGGAA	
hTR 3′_Fw	AGTTCGCTTTCCTGTTGGTG	3′ extended hTR transcript
hTR 3′_Rv	AGGTTTGGGGGTTCACAAG	
U2_M+3′_Fw	CGCTTCTCGGCCTTTTGGC	Total U2 snRNA
U2_M+3′_Rv	GTGCACCGTTCCTGGAGGT	
U2 3′_Fw	AGGGAGGTGAGAGACGGTAG	3′extended U2 snRNA transcript
U2 3′_Rv	GTGGAAACGAAAGCGTGCC	

**Table A3:** Quantity of RNA extracted during cell lysis and fractionation by RSBG40.

Content of Nonidet P-40 in RSBG40	RNA Content, %		
	Cytoplasmic Fraction	Nuclear Fraction	Nuclear Wash
0.5%	23%	55%	22%
1%	83%	13%	4%
